# Predictive Performance of Machine Learning for Suicide in Adolescents: Systematic Review and Meta-Analysis

**DOI:** 10.2196/73052

**Published:** 2025-06-16

**Authors:** Lingjiang Liu, Zhiyuan Li, Yaxin Hu, Chunyou Li, Shuhan He, Shibei Zhang, Jie Gao, Huaiyi Zhu, Guoping Huang

**Affiliations:** 1 Department of Psychiatry North Sichuan Medical College Nanchong China; 2 Sichuan Mental Health Center Department of Psychiatry The Third Hospital of Mianyang Mianyang China; 3 Department of Clinical Medicine Southwest Medical University Luzhou China

**Keywords:** machine learning, predictive model, meta-analysis, suicide prediction, adolescent mental health, suicide prevention

## Abstract

**Background:**

In the context of escalating global mental health challenges, adolescent suicide has become a critical public health concern. In current clinical practices, considerable challenges are encountered in the early identification of suicide risk, as traditional assessment tools demonstrate limited predictive accuracy. Recent advancements in machine learning (ML) present promising solutions for risk prediction. However, comprehensive evaluations of their efficacy in adolescent populations remain insufficient.

**Objective:**

This study systematically assessed the performance of ML-based prediction models across various suicide-related behaviors in adolescents, aiming to establish an evidence-based foundation for the development of clinically applicable risk assessment tools.

**Methods:**

This review assessed ML for predicting adolescent suicide–related behaviors. PubMed, Embase, Cochrane, and Web of Science databases were rigorously searched until April 20, 2024, and a multivariate prediction model was employed to assess the risk of bias. The c-index was used as the primary outcome measure to conduct a meta-analysis on nonsuicidal self-injury (NSSI), suicidal ideation, suicide attempts, suicide attempts combined with suicidal ideation, and suicide attempts combined with NSSI, evaluating their accuracy in the validation set.

**Results:**

A total of 42 studies published from 2018 to 2024 were included, encompassing 104 distinct ML models and 1,408,375 adolescents aged 11 to 20 years. The combined area under the receiver operating characteristic curve values for ML models in predicting NSSI, suicidal ideation, suicide attempts, suicide attempts combined with suicidal ideation, and suicide attempts combined with NSSI were 0.79 (95% CI 0.72-0.86), 0.77 (95% CI 0.71-0.83), 0.84 (95% CI 0.83-0.86), 0.82 (95% CI 0.79-0.84), and 0.75 (95% CI 0.73-0.76), respectively. The ML models demonstrated the highest combined sensitivity for suicide attempt prediction, with a value of 0.80 (95% CI 0.75-0.84), and the highest combined specificity for NSSI prediction, with a value of 0.96 (95% CI 0.94-0.99).

**Conclusions:**

Our findings suggest that ML techniques exhibit promising predictive performance for forecasting suicide risk in adolescents, particularly in predicting suicide attempts. Notably, ensemble methods, such as random forest and extreme gradient boosting, showed superior performance across multiple outcome types. However, this study has several limitations, including the predominance of internal validation methods employed in the included literature, with few studies employing external validation, which may limit the generalizability of the results. Future research should incorporate larger and more diverse datasets and conduct external validation to improve the prediction capability of these models, ultimately contributing to the development of ML-based adolescent suicide risk prediction tools.

## Introduction

A World Health Organization report highlights that suicide has become a significant public health issue globally [1]. Suicide-related behaviors encompass a range of actions involving varying degrees of both physical and psychological self-inflicted harm, including nonsuicidal self-injury (NSSI) and suicidal behaviors (such as suicidal ideation and suicide attempts) [2,3]. NSSI refers to intentional damage to body tissue with no explicit suicidal intent [4]. However, NSSI is widely deemed as an important risk factor for a suicide attempt [5]. Suicidal ideation is characterized by thoughts of planning or considering suicide, while a suicide attempt means the act of committing suicide [6]. Research by Chiu et al [7] suggested that suicidal ideation often precedes suicidal plans, and these plans serve as precursors to suicide attempts, which may ultimately result in fatal outcomes. These forms of suicide-related behaviors not only lead to individual tragedies but also pose substantial threats to the social and psychological well-being and stability of communities [8].

As this problem continues to escalate, suicide and suicide-related behaviors arising from psychological issues have become major public health concerns in the adolescent population [9]. Adolescence is a crucial phase for social and psychological growth [10]. During this time, due to immature psychological mechanisms, adolescents are highly susceptible to environmental influences, which may lead to psychological issues [11]. Kessler et al [12] argued that adolescence is a period when suicidal ideation and attempts are more likely to occur. Currently, early identification of high-risk adolescents is considered one of the most crucial strategies for preventing adolescent suicide [13]. However, Nock et al [14] noted that although suicidal ideation is relatively common among adolescents, most adolescents with such thoughts do not readily attempt suicide. Hall et al [15] found that many adolescents may not report their suicidal thoughts to parents or professionals early on and instead may confide in friends. This makes it difficult for families and professional institutions to identify individuals in need of help at an early stage. Given these challenges, it is important to know how to more effectively identify suicidal ideation or attempts, detect self-injurious behaviors, predict high-risk factors before the thoughts or actions emerge, and intervene promptly, in order to prevent and control suicide in adolescents. Moreover, it is becoming increasingly important to develop and popularize predictive models for adolescent suicide.

Currently, adolescent suicide prediction models mainly rely on psychological tests and surveys based on classic psychological and sociological theories. These traditional methods focus on specific indicators while overlooking other potentially crucial factors, such as social support and family environment. Although previous research [16] suggests that individuals with suicidal ideation or behaviors generally report higher scores on these surveys, this approach is heavily dependent on the subjective nature of questionnaire items, which limits its accuracy and predictive value [17]. Moreover, these questionnaires fail to account for the complex emotional and behavioral expressions of individuals in specific environments, thus failing to capture intricate nonlinear relationships. It is important to note that adolescents represent a unique population whose psychological and behavioral patterns significantly differ from those of adults. Many questionnaires have not adequately considered the specificities of different populations during their design and validation processes, resulting in variable effectiveness in practical applications. A groundbreaking meta-analysis quantitatively examined all suicide prediction models published over the past 50 years and concluded that these models have limited value in understanding and preventing suicide [18]. Concerning the concept of suicide itself, Belsher et al [19] suggested that the suboptimal performance of models in forecasting the risk of suicide, even in high-risk populations, is related to the low incidence of suicide. Franklin et al [18] also argued that the intricacy of suicide risk profiles makes it harder to forecast future suicidal events. Therefore, novel approaches are crucial for improving the accuracy of predicting adolescent suicidal behavior and understanding the underlying psychological mechanisms.

In recent years, machine learning (ML) and artificial intelligence techniques have shown potential in predicting the risk of suicide. Studies have leveraged these approaches to identify key risk factors for adolescent suicide and to enhance prediction accuracy. ML involves a set of algorithms exploring how computer systems learn rules from many examples without explicit programming [20]. By analyzing large datasets, ML can detect complex patterns and potential risk factors that may be overlooked by traditional models. Ehtemam et al [21] and Kirtley et al [22] suggested that this predictive approach could effectively reduce the incidence of suicide events. In clinical settings, ML models may assist health care professionals in the early identification of individuals at elevated risk for suicide, thereby enabling timely intervention. The introduction of ML models offers a new perspective and possibility for predicting the risk of suicide. ML encompasses a variety of modeling approaches, and the performance of different models may exhibit notable discrepancies. This variability presents a challenge in selecting the most suitable model for clinical practice. While some studies have employed ML models to predict adolescent suicide, few of these models have undergone external validation, despite this being a crucial step in advancing research on suicide risk prediction [23]. Furthermore, considering the particularly sensitive nature of mental health data in adolescents, these models have yet to be validated in larger, more diverse adolescent populations. This limits the ability to assess their applicability across different environments and cultural contexts. As such, the application of ML within adolescent populations remains devoid of robust, systematic, evidence-based support. In view of this, our study aims to comprehensively explore the performance and reliability of ML methods in forecasting the risk of adolescent suicide and to lay an evidence-based foundation for the future clinical applications of ML. Future research in this area should focus on optimizing feature selection, integrating multimodal data sources, and conducting prospective validation. Such advancements will enable the development of more efficient and reliable clinical tools, thereby increasing predictive accuracy and improving medical decision-making.

## Methods

### Registration

Our study was prospectively registered on PROSPERO (ID: CRD42024566433). The study followed the PRISMA (Preferred Reporting Items for Systematic Reviews and Meta-Analyses) guidelines [24] (Multimedia Appendix 1).

### Eligibility Criteria

The inclusion criteria were as follows: (1) studies involving minors as the research subjects and (2) studies that developed a complete ML model for predicting suicide-related behaviors. Moreover, studies that performed only internal validation without external validation were also considered for inclusion in this systematic review.

The exclusion criteria were as follows: (1) conference abstracts, brief reports, or other publications that were not peer-reviewed, (2) studies only analyzing risk or predictive factors for suicide-related behaviors without a complete ML model, (3) studies with a sample size of fewer than 20 cases, and (4) studies that did not report any measures of model accuracy (receiver operating characteristic curve, sensitivity, specificity, confusion matrix, accuracy, *F*_1_-score, and calibration curve).

### Literature Retrieval

PubMed, Web of Science, Cochrane, and Embase databases were systematically searched up to April 20, 2024. The strategy involved both subject headings and free-text terms, covering terms such as “adolescent,” “suicide,” and “ML.” To ensure global representativeness and the quality of the research, no regional or time-related restrictions were imposed. The detailed search strategy is illustrated in Multimedia Appendix 2.

### Literature Screening

The selection and screening of studies were independently performed by 2 researchers (LL and ZL). In cases of discrepancies during the screening process, consensus was reached by team discussion or consultation with a third expert (GH). The retrieved articles were uploaded to EndNote. After duplicates were removed, the titles and abstracts of the remaining articles were checked. Full-text articles were downloaded and further screened to obtain eligible articles. Additionally, references to the selected articles were examined to identify any potentially missed articles.

### Data Extraction

A standardized data extraction spreadsheet was used to collect key information, including the first author, publication year, author country, study type, patient source, population, predicted event, event definition, number of predicted events, total cases, number of predicted events in the training or validation set, total cases in the training or validation set, method of validation set generation, overfitting method, prediction settings for event number, validation set case number, missing data handling method, variable selection method, model type, and modeling variables. Data extraction was performed independently by 2 researchers (LL and ZL) who cross-checked their work. Disputes, if any, were settled by team discussion or consultation with a third expert (GH).

### Risk of Bias Assessment

PROBAST (Prediction Model Risk of Bias Assessment Tool), a standard quality evaluation tool [25], was employed to assess the risk of bias in models from the original studies. This tool comprises a series of questions across 4 distinct domains: participant, predictor, outcome, and statistical analysis, presenting the overall risk of bias and applicability. These domains include 2, 3, 6, and 9 questions, respectively, each of which can be answered in 1 of 3 ways: yes/possibly yes, no/possibly no, or no information. A domain was considered at high risk if at least one question was answered with “no” or “possibly no.” A domain was considered at low risk if all questions were answered with “yes” or “possibly yes.” The overall risk of bias was deemed low if all domains were rated as low risk, while the overall risk was considered high if at least one domain was rated as high risk. Two researchers (LL and YH) independently appraised the risk of bias based on PROBAST by carefully reviewing the methodological section of each paper. Following this, cross-checking was performed, and in cases of disagreement, a third researcher (GH) was consulted for determination.

### Definition of Outcome Events

In this study, suicide events were categorized into 5 subgroups: NSSI, suicide attempts, suicidal ideation, suicide attempts combined with suicidal ideation, and suicide attempts combined with NSSI. Subgroup analyses of the ML models based on the type of predicted event were performed to examine the impact of specific events on effect sizes.

### Statistical Analysis

A meta-analysis was performed on the area under the receiver operating characteristic curve (AUC), a metric for evaluating the overall accuracy of ML models. In certain primary studies where the c-index lacked 95% CI and standard error, the standard error was estimated based on the work of Debray et al [26]. Heterogeneity across studies was assessed via the *I*² statistic. When the *I*² value was >50%, a random effects model was adopted to summarize the AUC, and when the *I²* value was <50%, a fixed effects model was used. A meta-analysis was conducted solely on the validation set of the models. We have discussed the performance of ML in forecasting NSSI, suicide attempts, suicidal ideation, suicide attempts combined with suicidal ideation, and suicide attempts combined with NSSI.

Moreover, a meta-analysis was conducted on the sensitivity and specificity of ML in predicting suicide-related events using diagnostic 2×2 tables. A bivariate mixed effects model was employed. In cases where many primary studies did not provide 2×2 tables, the sensitivity, specificity, precision, and accuracy data provided in the original studies, along with case numbers, were employed to estimate the diagnostic 2×2 table. A *P* value of <.05 was considered to indicate statistical significance.

## Results

### Literature Screening

**Figure 1:**
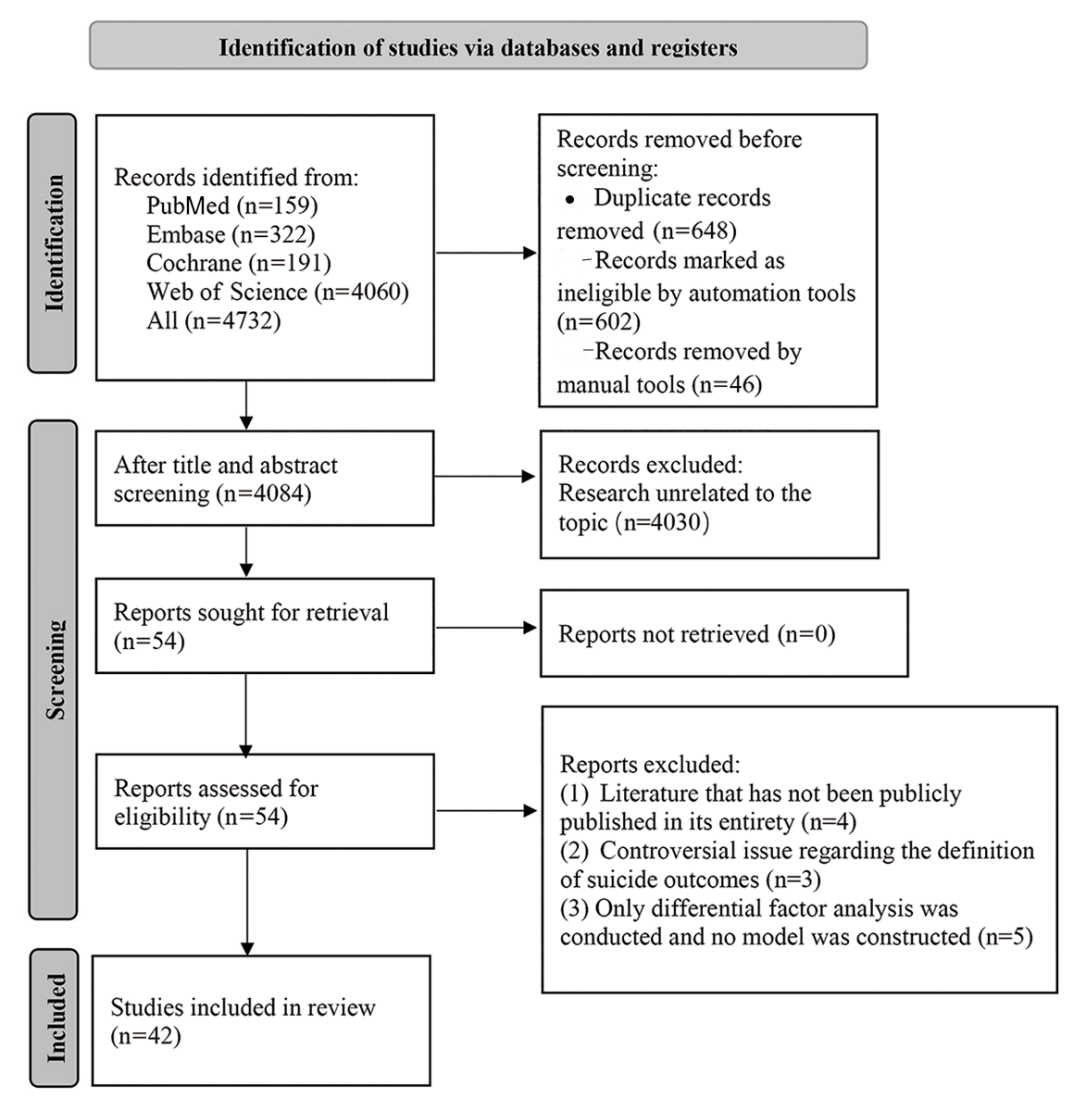
Literature screening process.

This study initially identified 4732 published articles, and 648 duplicate references were discarded automatically and manually via EndNote. The titles and abstracts of the remaining 4084 articles were reviewed, and 4030 articles were deleted, including studies unrelated to the research topic and ineligible studies, such as reviews, guidelines, conference abstracts, case reports, replies, and letters. For potentially eligible studies, the full texts were obtained and carefully evaluated. After a full-text review, 12 articles were removed. Of these 12 articles, 4 were unpublished in full, 3 involved controversial definitions of suicide outcomes, and 5 conducted only differential factor analyses without constructing models. Ultimately, 42 articles [27-68] were included in this study (Figure 1).

### Description of Basic Characteristics

The 42 articles included 1,408,375 adolescents aged 11 to 20 years. While there were slight variations in the age groups across different studies, the overall coverage encompassed the critical stages from adolescence to early adulthood. The analysis incorporated 104 distinct types of ML models from articles published between 2018 and 2024. Among the studies, 23 involved single-center data, 11 involved multi-center data, and 8 involved registry databases. The studies covered various countries and regions, including North America, Europe, and Asia, with a higher number of articles from the United States (n=11), South Korea (n=9), and China (n=8). The types of studies in this review were diverse, and there were 9 cross-sectional studies, 22 prospective cohort studies, 7 retrospective cohort studies, and 4 case-control studies. Among the studies, 16 considered suicide attempts combined with suicidal ideation as the outcome event, 9 focused on suicide attempts, and 7 focused on suicidal ideation. Moreover, 7 studies regarded NSSI as a predictive event for suicide, and 3 combined NSSI with suicide attempts. Most studies employed internal validation, with k-fold cross-validation used in 35 studies and bootstrapping used in 2 studies. Only 5 studies performed external validation. The most common validation methods used were k-fold cross-validation and bootstrap sampling to prevent model overfitting. Of the 42 studies, 37 performed multivariable analysis to screen for high-risk factors and 5 employed both univariate and multivariate analyses. The ML algorithms included support vector machine (SVM), random forest (RF), artificial neural network (ANN), logistic regression (LR), gradient boosting machine (GBM), decision tree (DT), and extreme gradient boosting (XGBoost) (Table 1).

**Table 1:** Basic characteristics of the included studies.

Study	Country	Study type	Patient source	Participants	Predicted events	Number of event cases	Total number of cases	Total number of cases in the training set	Method for generation of the validation set	Total number of cases in the validation set	Model type
Walsh et al [27], 2018	United States	Retrospective cohort study	Registry database	Adolescents aged <18 years	NSSI^a^	974	1470	1470	Bootstrapping + optimism adjustment	1470	LR^b^ and RF^c^
Jung et al [28], 2019	South Korea	Retrospective cohort study	Multicenter	Junior high school students in grades 1-3 and high school students in grades 1-3	Suicide attempts and suicidal ideation	7443	59,984	59,984	5-fold cross-validation	7443	LR, RF, SVM^d^, ANN^e^, and XGBoost^f^
Burke et al [29], 2020	United States	Retrospective cohort study	Multicenter	Adolescents presenting to emergency departments and primary care clinics	NSSI	608; emergency department samples: 1113; primary care samples: 608	Emergency department samples: 13,325; primary care samples: 12,001	Emergency department samples: 834; primary care samples: 456	Bootstrapping	Emergency department samples: 278; primary care samples: 152	Decision tree, RF, and ridge regression
Hill et al [30], 2019	United States	Retrospective cohort study	Registry database	Adolescents with a mean age of 15 years	Suicide attempts	192	4834	—^g^	10-fold cross-validation	—	Classification trees
Iorfino et al [31], 2020	Australia	Retrospective cohort study	Single center	Adolescents at the first visit to doctors	Suicide attempts and NSSI	320	1962	288	10-fold cross-validation	32	RF, Boruta, lasso regression, elastic net regression, Bayesian additive regression trees, and LR
Miché et al [32], 2019	Switzerland	Prospective cohort study	Single center	Adolescents in the community	Suicide attempts and suicidal ideation	137	2793	123	10-fold cross-validation	14	LR, lasso regression, ridge regression, and RF
Su et al [33], 2020	United States	Retrospective cohort study	Registry database	Adolescents at risk for suicide	Suicide attempts and suicidal ideation	180	41,721	162	10-fold cross-validation	18	Logistic regression classifier
Ballester et al [34], 2021	Canada	Prospective cohort study	Single center	Young people between 18 and 24 years old	Suicide attempts and suicidal ideation	91	1069	1069	4-fold cross-validation	23	XGBoost
Choi et al [35], 2021	South Korea	Retrospective cohort study	Multicenter	Adolescents who participated in the mental status examination	Suicidal ideation	306	31,720	—	10-fold cross-validation + external validation	—	LR, SVM, and graph isomorphism network
Kim et al [36], 2021	South Korea	Retrospective cohort study	Multicenter	Children and adolescents aged 12-17 years receiving outpatient psychiatric care	Suicide attempts and suicidal ideation	44	124	124	10-fold cross-validation	—	LR, RF, ANN, SVM, and XGBoost
Lekkas et al [37], 2021	United States	Retrospective cohort study	Single center	Individuals with acute suicidal thoughts	Suicidal ideation	—	47	—	10-fold cross-validation	—	XGBoost, logit boost, generalized linear models via penalized maximum likelihood (glmnet), k-nearest neighbors, 3-layered feed-forward neural networks, aggregating and averaging randomly seeded neural networks (avnnet), and naive Bayes classifier
Macalli et al [38], 2021	France	Prospective cohort study	Single center	Students aged 18 years or older in the i-Share project	Suicidal ideation	874	5066	—	10-fold cross-validation	—	RF
Mouchabac et al [39], 2021	France	Retrospective cohort study	Single center	Adolescent patients	Suicide attempts and suicidal ideation	—	—	—	10-fold cross-validation	—	Bayesian network model
Navarro et al [40], 2021	Canada	Retrospective cohort study	Registry database	Adolescents born in Quebec, Canada	Suicide attempts	134	1623	—	10-fold cross-validation	—	RF
van Vuuren et al [41], 2021	Netherlands	Prospective cohort study	Single center	Second and fourth grade students at a secondary school in Amsterdam, the Netherlands	Suicide attempts and suicidal ideation	—	—	—	10-fold cross-validation	—	RF and LASSO^h^
Weller et al [67], 2021	United States	Retrospective cohort study	Single center	High school students in the state of Utah	Suicide attempts and suicidal ideation	—	174,864	110,391	10-fold cross-validation	13,629	K-nearest neighbor algorithm, naive Bayes, LR, decision tree classifier, XGBoost, and LightGBM
Fonseca-Pedrero et al [42], 2022	Spain	Cross-sectional study	Single center	Students in La Rioja, northern Spain	Suicide attempts and suicidal ideation	—	1790	—	10-fold cross-validation	—	—
Huang et al [43], 2022	China	Cross-sectional study	Single center	Chinese teenagers in the first year of junior high school and high school	Suicidal ideation	—	10,243	—	10-fold cross-validation	—	RF, SVM, and decision tree
Lim et al [68], 2022	South Korea	Retrospective cohort study	Registry database	Adolescents between 12 and 18 years old	Suicide attempts and suicidal ideation	287,104	410,147	—	10-fold cross-validation + external validation	123,043	LR, RF, ANN, SVM, and XGBoost
Zheng et al [44], 2022	United States	Case-control study	Single center	Public high school students in the state of Mississippi 2001-2019	Suicide attempts and suicidal ideation	—	—	—	10-fold cross-validation	—	RF, SVM, and neural network
Park et al [45], 2022	South Korea	Case-control study	Multicenter	Middle and high school students in South Korea	Suicidal ideation	5979	54,948	—	10-fold cross-validation	—	XGBoost
Yang et al [46], 2022	China	Case-control study	Single center	Adolescents aged 10-24 years with mood disorders	NSSI	137	186	—	10-fold cross-validation	—	SVM
Bajaj et al [47], 2023	United States	Retrospective cohort study	Single center	Adolescents at risk for suicide and normally growing	Suicide attempts and suicidal ideation	—	—	—	10-fold cross-validation	—	SVM
Czyz et al [48], 2021	United States	Prospective cohort study	Single center	Adolescent psychiatric inpatients who want to commit suicide (13-17 years old)	Suicidal ideation	—	—	—	10-fold cross-validation	—	Multi-level classification and regression tree
Donnelly et al [49], 2023	South Korea	Cross-sectional study	Single center	Middle school students in Korea	Suicidal ideation	—	6666	—	10-fold cross-validation	—	Decision tree, LR, and naive Bayes classifier
Gossage et al [50], 2023	New Zealand	Cross-sectional study	Single center	Adolescents of Pacific islanders	Suicide attempts	—	—	—	10-fold cross-validation	—	Network analysis
Haghish et al [51], 2023	Norway	Retrospective cohort study	Multicenter	Norwegian adolescents (13-18 years old)	Suicide attempts	8090	173,664	—	10-fold cross-validation	—	XGBoost
Kirlic et al [52], 2021	United States	Retrospective cohort study	Single center	356 undergraduates at a private university in the Midwest who did not seek treatment	Suicide attempts and suicidal ideation	—	—	356	10-fold cross-validation + external validation	—	Elastic network, support vector regression, RF, and k-nearest neighbor
Kwon et al [53], 2023	South Korea	Retrospective cohort study	Multicenter	Adolescents between 13 and 18 years old in Korea	Suicide attempts	1960	95,677	—	5-fold cross-validation + external validation	—	LR, SVM, RF, XGBoost
Haghish et al [54], 2023	Norway	Cross-sectional study	Multicenter	Adolescents between 13 and 18 years old in Norway	Suicide attempts	8075	173,664	138,931	10-fold cross-validation	34,733	Base-learner algorithm and stacked ensemble algorithm
Jankowsky et al [55], 2024	Germany	Prospective cohort study	Registry database	17-year-old adolescents	Suicide attempts	502	7347	—	10-fold cross-validation	—	Elastic net regression, GBM^i^, and LR
Gholi Zadeh Kharrat et al [56], 2024	Canada	Case-control study	Registry database	Adolescents at risk for suicide	Suicide attempts and suicidal ideation	18,339	20,339	9440	10-fold cross-validation	8899	LR, RF, XGBoost, and multilayer perceptron
Lee et al [57], 2024	South Korea	Retrospective cohort study	Registry database	Korean adolescents aged 13 to 18 years with allergic rhinitis	Suicide attempts	—	KYRBS dataset: 299,468 samples; KNHANES dataset: 833 samples	—	10-fold cross-validation + external validation	833	RF, XGBoost, AdaBoost, and light GBM
Mürner-Lavanchy et al [58], 2024	Switzerland	Cross-sectional study	Single center	Adolescents with NSSI	NSSI	—	149	—	10-fold cross-validation	—	LR, elastic net regression, RF, gradient lifting tree
Wang et al [59], 2024	China	Retrospective cohort study	Multicenter	Students at universities in Northeast China	NSSI	4976	95,833	—	10-fold cross-validation	—	Chi-squared automatic interaction detector model
Wang et al [60], 2024	China	Retrospective cohort study	Multicenter	Depressed adolescents between 12 and 18 years	Suicide attempts and suicidal ideation	2154	2300	—	10-fold cross-validation	—	Decision tree
Wang et al [61], 2024	China	Prospective cohort study	Single center	Students from a junior high school in Guangdong Province	Suicide attempts and suicidal ideation	348	1750	—	10-fold cross-validation	—	RF
Zhong et al [62], 2024	China	Retrospective cohort study	Multicenter	Adolescents aged 10-19 years from 50 schools in western Sichuan	NSSI	617	13,304	9313	10-fold cross-validation	3991	XGBoost
Zhou et al [63], 2024	China	Cross-sectional study	Single center	Middle school students from 8 middle schools in Wuhan, China	NSSI	791	7967	5949	10-fold cross-validation	2018	RF and LR
Lee et al [64], 2021	South Korea	Cross-sectional study	Single center	—	Suicide attempts and NSSI	—	1731	—	10-fold cross-validation	—	—
Su et al [65], 2023	Australia	Cross-sectional study	Single center	—	Suicide attempts and NSSI	—	2809	—	10-fold cross-validation	—	—
Sara et al [66], 2024	Bangladesh	Retrospective cohort study	Single center	—	Suicide attempts	—	584	—	10-fold cross-validation	—	—

^a^NSSI: nonsuicidal self-injury.

^b^LR: logistic regression.

^c^RF: random forest.

^d^SVM: support vector machine.

^e^ANN: artificial neural network.

^f^XGBoost: extreme gradient boosting.

^g^Not available or not applicable.

^h^LASSO: least absolute shrinkage and selection operator.

^i^GBM: gradient boosting machine.

### Risk of Bias Assessment

**Figure 2:**
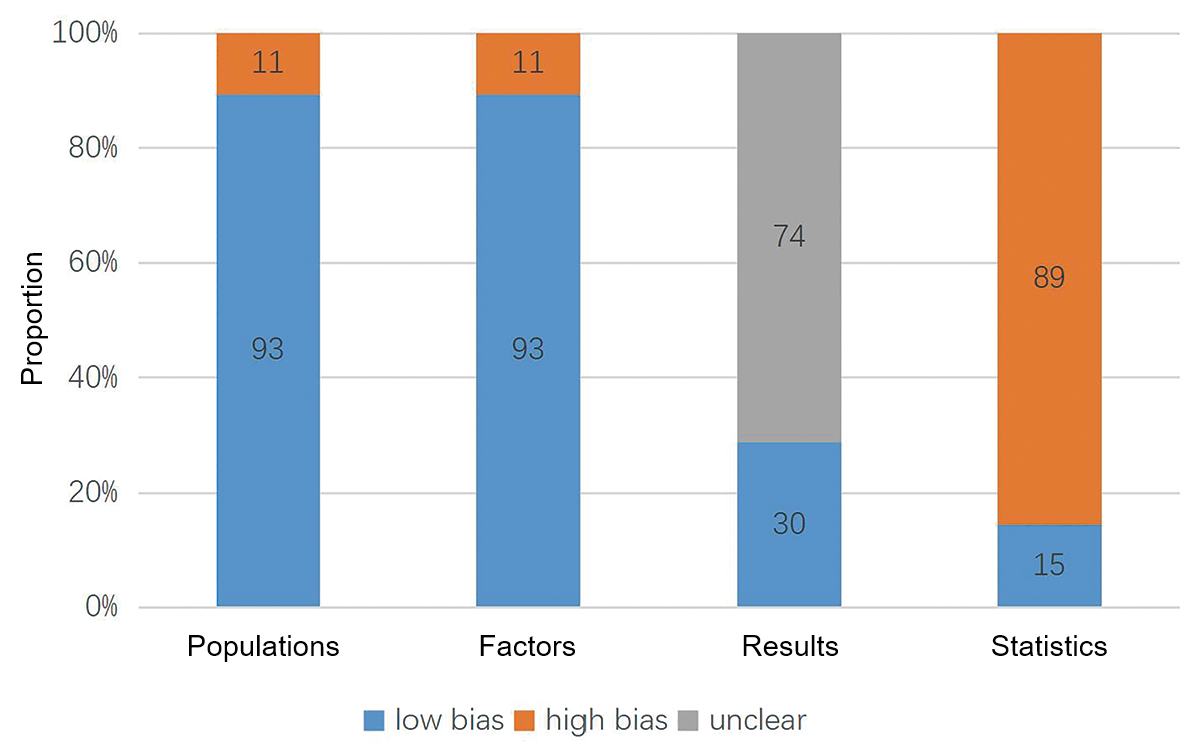
Risk of bias of suicide prediction models (N=104).

The risk of bias in 104 suicide prediction models was assessed. Of these 104 models, 92 (88.5%) were deemed to have a high risk of bias and 12 (11.5%) were deemed to have a low risk of bias. Models with a high risk of bias exhibited significant discrepancies in 4 different domains. Among the models, 11 (10.6%) had a high risk of bias in the research subject domain, 11 (10.6%) had a high risk of bias in the predictor domain, 0 (0%) had a high risk of bias in the outcome domain, and 89 (85.6%) had a high risk of bias in the statistical analysis domain (Figure 2). Within the statistical analysis domain, the most common causes of an elevated risk of bias were unreasonable sample sizes (62/104, 59.6%) and improper handling of continuous and categorical independent variables (78/104, 75.0%). These findings indicate that some studies may have flaws in terms of design and lack transparency in methodology and data reliability, which could undercut the accuracy and reliability of their conclusions.

### Meta-Analysis Results

#### NSSI Findings

Five types of models predicted NSSI, with a combined AUC of 0.79 (95% CI 0.72-0.86) for predicting NSSI behavior. Subgroup analysis by different ML models revealed that the RF model demonstrated the strongest predictive ability, with an effect size of 0.86 (95% CI 0.78-0.94). The combined sensitivity for predicting NSSI was 0.51 (95% CI 0.41-0.61), and the combined specificity was 0.96 (95% CI 0.94-0.99). The RF model also exhibited the highest sensitivity and specificity at 0.74 (95% CI 0.53-0.88) and 0.99 (95% CI 0.28-1.00), respectively (Tables 2 and 3; Multimedia Appendix 3).

**Table 2:** Meta-analysis results of the c-index of machine learning models predicting suicide.

Outcome events and models			Number of models		AUC^a^, value (95% CI)	
**Nonsuicidal self-injury**			14		0.79 (0.72-0.86)	
	Random forest	4		0.86 (0.78-0.94)		
	Logistic regression	4		0.81 (0.77-0.86)		
	Artificial neural network	1		0.70 (0.64-0.76)		
	Gradient boosting machine	1		0.67 (0.61-0.73)		
	Decision tree	2		0.68 (0.65-0.70)		
**Suicide attempts**			24		0.84 (0.83-0.86)	
	Random forest	7		0.81 (0.77-0.84)		
	Artificial neural network	1		0.83 (0.68-0.98)		
	Gradient boosting machine	4		0.85 (0.82-0.89)		
	Extreme gradient boosting	6		0.87 (0.84-0.91)		
	Light gradient boosting machine	4		0.80 (0.82-0.89)		
	Adaptive boosting	4		0.86 (0.82-0.89)		
**Suicide attempts combined with suicidal ideation**			37		0.82 (0.79-0.84)	
	Random forest	9		0.82 (0.77-0.88)		
	Logistic regression	7		0.80 (0.73-0.88)		
	Artificial neural network	5		0.86 (0.81-0.92)		
	Decision tree	1		0.64 (0.61-0.67)		
	Extreme gradient boosting	8		0.82 (0.76-0.88)		
	Light gradient boosting machine	1		0.91 (0.90-0.92)		
	Support vector machine	2		0.90 (0.81-0.98)		
	Naive Bayes	1		0.52 (0.51-0.53)		
	K-nearest neighbor	1		0.84 (0.83-0.85)		
**Suicidal ideation**			17		0.77 (0.71-0.83)	
	Random forest	3		0.86 (0.81-0.92)		
	Logistic regression	1		0.74 (0.71-0.77)		
	Support vector machine	1		0.57 (0.55-0.60)		
	K-nearest neighbor	2		0.74 (0.32-1.16)		
	Artificial neural network	2		0.69 (0.64-0.74)		
	Naive Bayes	1		0.70 (0.63-0.78)		
	CatBoost	1		0.89 (0.85-0.92)		
	Decision tree	4		0.79 (0.68-0.90)		
	Extreme gradient boosting	2		0.78 (0.50-1.06)		
**Suicide attempts combined with nonsuicidal self-injury**			5		0.75 (0.73-0.76)	
	Random forest	3		0.75 (0.72-0.77)		
	Logistic regression	1		0.75 (0.72-0.78)		
	Artificial neural network	1		0.75 (0.72-0.78)		

^a^AUC: area under the receiver operating characteristic curve.

**Table 3:** Meta-analysis results of the sensitivity and specificity of machine learning models predicting suicide.

Outcome events and models			Number of models		Sensitivity^a^, value (95% CI)		Specificity^a^, value (95% CI)	
**Nonsuicidal self-injury**			10		0.51 (0.41-0.61)		0.96 (0.94-0.99)	
	Random forest	4		0.74 (0.53-0.88)		0.99 (0.28-1.00)		
	Logistic regression	2		(0.49-0.81)		(0.77-0.81)		
	Artificial neural network	1		0.45		0.81		
	Gradient boosting machine	1		0.51		0.76		
	Decision tree	2		(0.02-0.04)		(0.99-1.00)		
**Suicide attempts**			23		0.80 (0.75-0.84)		0.83 (0.80-0.85)	
	Random forest	5		0.80 (0.68-0.88)		0.82 (0.75-0.87)		
	Decision tree	2		(0.70-0.91)		(0.71-0.86)		
	Artificial neural network	1		0.88		0.88		
	Gradient boosting machine	1		0.89		0.89		
	Extreme gradient boosting	6		0.81 (0.71-0.88)		0.84 (0.77-0.89)		
	Light gradient boosting machine	4		0.72 (0.65-0.78)		0.82 (0.74-0.88)		
	Adaptive boosting	4		0.72 (0.62-0.79)		0.82 (0.74-0.87)		
**Suicide attempts combined with suicidal ideation**			38		0.58 (0.52-0.64)		0.93 (0.90-0.95)	
	Random forest	9		0.52 (0.41-0.63)		0.92 (0.86-0.96)		
	Logistic regression	6		0.54 (0.40-0.68)		0.94 (0.87-0.98)		
	Artificial neural network	6		0.59 (0.45-0.72)		0.95 (0.89-0.98)		
	Decision tree	1		0.34		0.94		
	Extreme gradient boosting	9		0.59 (0.47-0.71)		0.92 (0.82-0.96)		
	Light gradient boosting machine	1		0.85		0.94		
	K-nearest neighbor	1		0.68		0.90		
	LASSO^b^ regression	1		0.52		0.85		
	Naive Bayes	1		0.34		0.80		
	Support vector machine	3		(0.64-0.85)		(0.79-0.96)		
**Suicidal ideation**			23					
	Random forest	3		(0.75-0.81)		(0.71-0.93)		
	Logistic regression	1		0.50		0.98		
	CatBoost	1		0.72		0.92		
	K-nearest neighbor	2		(0.19-0.70)		(0.92-0.98)		
	Artificial neural network	2		0.76		(0.69-0.73)		
	Naive Bayes	1		0.77		0.65		
	Support vector machine	1		0.15		1.00		
	Decision tree	4		0.86 (0.77-0.92)		0.89 (0.83-0.93)		
	Extreme gradient boosting	2		(0.62-0.66)		(0.69-0.91)		
**Suicide attempts combined with nonsuicidal self-injury**			6		0.63 (0.52-0.74)		0.78 (0.69-0.85)	
	Random forest	3		(0.36-0.68)		(0.72-0.91)		
	Logistic regression	2		(0.70-0.75)		(0.68-0.69)		
	Artificial neural network	1		0.74 (0.62-0.79)		0.72 (0.63-0.78)		

^a^In the case of only 2-3 diagnostic 4-grid tables, only ranges (CIs) are provided, while in the case of only 1 diagnostic 4-grid table, only exact values (point estimations) are provided.

^b^LASSO: least absolute shrinkage and selection operator.

#### Suicide Attempts

Seven types of models predicted suicide attempts, with the combined AUC for ML methods being 0.84 (95% CI 0.83-0.86). The XGBoost model exhibited the strongest predictive ability, with an AUC of 0.87 (95% CI 0.84-0.91). The combined sensitivity for predicting suicide attempts was 0.80 (95% CI 0.75-0.84), and the combined specificity was 0.83 (95% CI 0.80-0.85). The XGBoost model also showed the highest sensitivity and specificity at 0.81 (95% CI 0.71-0.88) and 0.84 (95% CI 0.77-0.89), respectively (Tables 2 and 3; Multimedia Appendix 3).

#### Suicidal Ideation

Nine types of models predicted suicidal ideation, with the combined AUC for ML methods being 0.77 (95% CI 0.71-0.83). The CatBoost model demonstrated the strongest predictive ability, with an AUC of 0.89 (95% CI 0.85-0.92). The combined sensitivity for predicting suicidal ideation was 0.71 (95% CI 0.64-0.79), and the combined specificity was 0.89 (95% CI 0.85-0.93). The DT model exhibited the highest sensitivity and specificity at 0.86 (95% CI 0.77-0.92) and 0.89 (95% CI 0.83-0.93), respectively (Tables 2 and 3; Multimedia Appendix 3).

#### Suicide Attempts Combined With Suicidal Ideation

The largest number of model types (n=10) predicted suicide attempts combined with suicidal ideation, with a combined AUC of 0.82 (95% CI 0.79-0.84). Among these, the LightGBM model exhibited the strongest predictive ability, with an AUC of 0.91 (95% CI 0.90-0.92). The combined sensitivity for predicting suicide attempts combined with suicidal ideation was 0.58 (95% CI 0.52-0.64), and the combined specificity was 0.93 (95% CI 0.90-0.95). The LightGBM model exhibited the highest sensitivity at 0.85 (95% CI 0.47-0.71), while the ANN model demonstrated the highest specificity at 0.95 (95% CI 0.89-0.98) (Tables 2 and 3; Multimedia Appendix 3).

#### Suicide Attempts Combined With NSSI

Only 3 types of models predicted suicide attempts combined with NSSI, with a combined AUC of 0.75 (95% CI 0.73-0.76). The RF model exhibited the best predictive ability, with an AUC of 0.76 (95% CI 0.72-0.78). The combined sensitivity for predicting suicide attempts combined with NSSI was 0.63 (95% CI 0.52-0.74), and the combined specificity was 0.78 (95% CI 0.69-0.85). The ANN model exhibited the highest sensitivity and specificity at 0.74 (95% CI 0.62-0.79) and 0.72 (95% CI 0.63-0.78), respectively (Tables 2 and 3; Multimedia Appendix 3).

## Discussion

### Summary of Key Findings

This systematic review and meta-analysis was carried out to evaluate the performance of ML in predicting the risk of suicide in adolescents. The goal was to provide a more precise risk assessment tool to assist clinicians and public health professionals in preventing suicide among adolescents. Through model interpretation and validation, ML techniques demonstrated considerable potential in predicting the risk of suicide in adolescents.

Despite the increasing focus on adolescent suicide crisis interventions in recent years, there remains a lack of consensus on how to best conceptualize these behaviors. One of the most debated issues in the existing literature is whether NSSI should be classified as a suicide-related behavior. Some studies argue that suicide attempts and NSSI are distinct, while others contend that they are complementary and strongly interconnected, both falling under the category of suicide-related behaviors [69-74]. Klonsky et al [73] acknowledged a strong correlation between NSSI and suicide attempts. A survey of adolescents in Scotland found that at least one-ninth of interviewees reported a suicide attempt, 1 in 6 reported NSSI, and 6.5% reported both behaviors [74], indicating a significant interaction between them. Ultimately, this review identified 6 studies on NSSI, and 3 studies predicted both NSSI and suicide attempts. From the perspective of preventing suicide risk, this review categorizes NSSI into suicide-related behaviors, aiming to encompass all behaviors that may potentially lead to suicidal outcomes.

### Comparison With Other Literature

ML techniques have increasingly been applied to predict suicide risk. Researchers have continuously improved the accuracy of ML in predicting the risk of suicide through multiple iterations. In recent years, several studies have examined the performance of ML models in predicting suicide-related outcomes. Studies by Wang et al [75] and Kusuma et al [76] indicate that ML can surpass traditional clinical and statistical methods, providing higher prediction accuracy. In this review, the AUC values of all models predicting various suicide outcome events exceeded 50%, and the vast majority of AUC values stabilized between 70% and 80%, demonstrating the strong discriminative power of ML models in predicting suicide-related events. The results of random effects models revealed that ML algorithms also exhibited high sensitivity and specificity in predicting suicide-related behaviors, showing clear superiority. Ehtemam et al [21] and Corke et al [77], in their reported reviews, compared ML with traditional models, focusing on the predictive value of ML models for the risk of suicide. Kusuma et al [76] used more appropriate metrics to assess the performance of ML models in the prediction of suicide-related outcomes, including ideation, attempts, and mortality. However, these studies generally relied on selected algorithms, such as RF and LR, without sufficiently justifying the rationale for these choices or systematically comparing a broader range of algorithms. As a result, there may be an overreliance on specific types of ML techniques, potentially overlooking alternative algorithms that may be more suited to particular datasets or research questions. In this context, this study, by comparing the predictive performance of various ML models, seeks to present valuable insights for selecting predictive models for different suicide outcomes.

Another notable limitation lies in the integration of diverse datasets. In most previous literature, the robustness of ML predictions often depended on the availability of large-scale, high-quality datasets. Many of the samples in these studies failed to adequately represent the broader population characteristics, thereby limiting the generalizability of their findings. Currently, no systematic review or meta-analysis has specifically focused on the prediction of suicide risk in adolescent populations as a high-risk group. This gap may stem from the limited quality and generalizability of adolescent sample data. Among the included studies on prediction models for adolescent suicide, over 50% (23 studies) used single-center data. Samaga et al [78] argued that the homogeneity of such data limits the model’s ability to learn broader features, thereby impacting its adaptability to different contexts. Furthermore, adolescent samples are often affected by issues, such as sample homogeneity, selection bias, self-reporting bias, background differences in samples, and sample attrition in longitudinal studies [79-83]. Hence, a far greater proportion of studies focus on the general population rather than adolescents as a specific research subject. Moreover, since suicide-related issues are sensitive, especially during the collection and sharing of adolescent data, which is often restricted by legal and ethical limitations, data availability is constrained. Researchers must not only face the challenges of societal sensitivity but also protect the private and personal information of participants. Obtaining sufficient suicide-related data is becoming increasingly difficult. Even when data are available, the quality of the data may be compromised due to cognitive limitations of adolescents, data generalizability issues, and potential reporting and collection errors. Through systematic reviews and meta-analyses integrating the results of all studies on the prediction of adolescent suicide, the accuracy and predictive performance of these models can be further enhanced.

### Comparison Among Models

In previous clinical studies based on ML, the types of models were generally simple, and traditional models, such as LR and DT, were predominant. However, among the studies included in our research, most used multiple ML approaches. This suggests that research on adolescent suicide tends to favor more complex and diverse ML techniques, particularly ensemble methods and nonlinear models. Furthermore, predicting suicide-related outcomes in adolescents using ML algorithms remains an emerging field of research. All the studies included in our analysis were conducted within the past 6 years, despite no restriction on the publication time during the literature search process. In our study, RF was the most commonly used ML model. It demonstrated notable superiority in handling imbalanced datasets, with an AUC value exceeding 80% in all 4 subgroups. A wealth of literature has also confirmed that the RF model, due to its ability to model nonlinear relationships and its insensitivity to overfitting, typically exhibits high predictive performance compared to other ML methods [84-86]. The XGBoost model also performed excellently, particularly in predicting suicide attempts, where it outperformed all other ML models with an AUC value of 0.87. In predicting other suicide-related events, the AUC value of XGBoost remained consistently around 0.8, which aligns with the findings of Ehtemam et al [21]. Additionally, Ehtemam et al [21] observed that neural network algorithms were less accurate. Unfortunately, our study did not compute the combined accuracy of this model. Hence, it is impossible to verify their findings. Nevertheless, the performance of neural networks in predicting certain suicide events in our study was impressive. In predicting suicide attempts and suicide attempts combined with suicidal ideation, ANN achieved an AUC of more than 80%. We believe that neural networks, which can handle complex nonlinear relationships, may have particular advantages in processing data related to mental health. Moreover, the AUC value of the LightGBM model was even more than 90% in the prediction of suicide attempts combined with suicidal ideation.

Regarding sensitivity and specificity, various ML models demonstrated relatively consistent results. Most models with higher sensitivity also exhibited higher specificity, with specificity higher than sensitivity. In this study, the ML model demonstrated the highest level of overall sensitivity in predicting suicide attempts, indicating its superior ability to minimize false negatives, that is, effectively identifying individuals who truly experience suicide attempts. Conversely, it exhibited the highest level of overall specificity in identifying NSSI, suggesting its excellent performance in reducing false positives. In other words, it can accurately exclude individuals who do not engage in NSSI behavior. This meta-analysis found that in suicide event subgroups containing at least four models, the XGBoost model demonstrated superior sensitivity and specificity in predicting the risk of suicide among adolescent patients. In particular, it had the highest sensitivity and specificity in predicting suicide attempts, with a sensitivity of 0.81 (95% CI 0.71-0.88) and a specificity of 0.84 (95% CI 0.77-0.89). This result indicates that the model accurately identified 81% of individuals with suicide attempts and correctly identified 84% of those without suicide attempts. This superior performance can primarily be attributed to the model’s optimization strategies. First, XGBoost uses a gradient boosting algorithm to iteratively optimize the loss function, enabling more precise capture of nonlinear relationships within the data [21]. Moreover, XGBoost supports the customization of the loss function, which allows the model to adjust its optimization objectives according to the specific task, thereby enhancing both sensitivity and specificity [21]. It was also observed that ML models, such as LightGBM, GBM, and ANN, exhibited high sensitivity and specificity. However, their application in the prediction of suicide events remains relatively limited, suggesting that these models hold considerable potential in the prediction of adolescent suicide and are expected to be broadly used in the prediction of suicide.

The significant differences in the performances of different ML models in predicting suicide-related events may be attributed to the characteristics of the models themselves. Suicide-related data often contain complex psychological, social, and behavioral characteristics, and some models may be more skillful at capturing the complex relationships between these characteristics. In addition, the size and quality of the datasets included in different studies may also affect the performance of the models. Future research can use more interpretability techniques and combine research results from psychology and sociology to better understand the reasons for differences in model performance.

### Importance of Model Validation in ML

In ML research, selecting an appropriate validation set generation method is crucial. In this study, there were 2 main methods for generating validation sets: internal validation and external validation. Internal validation primarily assesses the internal validity of the predictive model through techniques such as random sampling, cross-validation, and bootstrapping. In contrast, external validation evaluates the model’s ability to generalize to populations and environments that have not been explored by the dataset. By applying the model to diverse external datasets, researchers can mitigate the potential bias introduced by the original training cohort. This process helps to validate the model across various clinical pathways and population characteristics, ensuring that it is not merely reflective of the specific conditions of the training data [21]. However, internal validation remains the dominant method for validating ML models, as data barriers often complicate the interpretation of a model’s generalizability. Combining internal and external validation methods provides a more comprehensive understanding of the model’s generalization ability. This issue is especially critical in suicide prediction, which involves rare events and requires highly accurate models with strong generalizability [87,88]. Unfortunately, most of the studies included in this review predominantly employed internal validation, with a few using external validation. This may result in misleadingly optimistic accuracy estimates, raising concerns about the model’s applicability in real-world settings.

### Strengths and Limitations

This study provides preliminary evidence for adolescent suicide and incorporates a sufficiently large sample size to ensure the robustness of the evidence. However, several limitations exist in our research. First, there was a notable degree of heterogeneity, which represents a common challenge faced by meta-analyses based on ML techniques. Subgroup analyses were carried out to explore the sources of this heterogeneity. Although most ML models demonstrated exceptional performance in identifying potential risks of suicide, the interpretability of their predictive results remains challenging. In the prediction of NSSI, suicidal ideation, suicide attempts, and combined suicidal ideation, there were considerable differences in model performance. The difference in the AUC of different models for predicting the same event was as large as 20%-30%. In contrast, all ML models exhibited high predictive efficacy for suicide attempts, with AUC values exceeding 80%. Glenn et al [89] suggested that ML models tend to perform better in predicting suicide attempts, which has a relatively low incidence rate. For the prediction of suicide attempts combined with NSSI, 3 models showed consistently good and balanced predictive performance.

Second, while the results from the validation sets were summarized, the validation methods of the models were predominantly internal validation approaches (such as k-fold cross-validation and the bootstrap method), which limits the generalizability of these models. Furthermore, since the number of eligible studies was limited, the psychological mechanisms underlying each suicide outcome or the predictive performances of ML for the risk of suicide in the context of socioeconomic factors across different suicide events were not thoroughly elucidated. Third, a 2×2 diagnostic contingency table was constructed based on sensitivity, specificity, positive predictive value, and accuracy, in conjunction with the total number of cases. The process of estimation, however, may introduce assumptions and estimation errors, potentially leading to bias in the meta-analysis. Finally, the ethical issues surrounding the use of ML for suicide prediction are often underestimated. ML models are typically regarded as “black boxes,” and concerns regarding the interpretability and transparency of their decision-making processes are particularly pronounced when predicting suicide risk. This is especially relevant when considering the potential clinical implications of such predictions [89]. Underestimating these concerns may pose risks, and if not managed appropriately, ML-driven clinical practices could inadvertently cause psychological harm to individuals or lead to societal consequences.

### Conclusion

Through a systematic review and meta-analysis, this study explored the reliability and accuracy of ML techniques in predicting the risk of suicide in adolescents. Our findings serve as a theoretical foundation and practical pathway for the future development of more precise and tailored prevention strategies. Our study also has limitations, including the lack of external validation and the potential for overfitting. Future research should incorporate larger and more diverse datasets, along with external validation, to enhance the practicality and reliability of prediction models. Future studies are expected to further advance the development of predictive models for adolescent suicide. Researchers can develop simple tools for predicting adolescent suicide risk based on ML methods, fostering interdisciplinary collaboration, and improving data collection methods, thereby enhancing the interpretability of these models. However, prior to the application of ML models in real-world settings, rigorous ethical review and adaptive adjustments are necessary to ensure that the implementation of these techniques does not negatively impact the safety and privacy of adolescents.

## Appendix

Multimedia Appendix 1 PRISMA (Preferred Reporting Items for Systematic Reviews and Meta-Analyses) checklist.

Multimedia Appendix 2 Literature search strategy.

Multimedia Appendix 3 Forest plot of the meta-analysis for the c-index of machine learning models predicting suicide-related events.

## Abbreviations

ANN: artificial neural network
AUC: area under the receiver operating characteristic curve
DT: decision tree
GBM: gradient boosting machine
LR: logistic regression
ML: machine learning
NSSI: nonsuicidal self-injury
PROBAST: Prediction Model Risk of Bias Assessment Tool
RF: random forest
SVM: support vector machine
XGBoost: extreme gradient boosting

## Data Availability

All data generated or analyzed during this study are included in this published article.
