# Supplementary material for: Predictive Performance of Machine Learning for Suicide in Adolescents: Systematic Review and Meta-Analysis
Source: J Med Internet Res. 2025 Jun 16;27:e73052. doi: 10.2196/73052 (PMC12209725; doi:10.2196/73052)
Supplement: Multimedia Appendix 2 [file jmir_v27i1e73052_app2.docx]

# Literature search strategy

**1.Pubmed**

| Search number | Query | Results |
| --- | --- | --- |
| #1 | "Adolescent"[Mesh] | 2,241,563 |
| #2 | (((((((((((((Adolescents[Title/Abstract]) OR (Adolescence[Title/Abstract])) OR (Teens[Title/Abstract])) OR (Teen[Title/Abstract])) OR (Teenagers[Title/Abstract])) OR (Teenager[Title/Abstract])) OR (Youth[Title/Abstract])) OR (Youths[Title/Abstract])) OR (child[Title/Abstract])) OR (youth[Title/Abstract])) OR (juvenile[Title/Abstract])) OR (children[Title/Abstract])) OR (minor[Title/Abstract])) OR (minors[Title/Abstract]) | 2,105,359 |
| #3 | ("Adolescent"[Mesh]) OR ((((((((((((((Adolescents[Title/Abstract]) OR (Adolescence[Title/Abstract])) OR (Teens[Title/Abstract])) OR (Teen[Title/Abstract])) OR (Teenagers[Title/Abstract])) OR (Teenager[Title/Abstract])) OR (Youth[Title/Abstract])) OR (Youths[Title/Abstract])) OR (child[Title/Abstract])) OR (youth[Title/Abstract])) OR (juvenile[Title/Abstract])) OR (children[Title/Abstract])) OR (minor[Title/Abstract])) OR (minors[Title/Abstract])) | 3,683,076 |
| #4 | machine learning[MeSH Terms] | 66,954 |
| #5 | ((((((((((((((((((((((((Transfer Learning[Title/Abstract]) OR (Deep learning[Title/Abstract])) OR (Ensemble Learning[Title/Abstract])) OR (artificial intelligence[Title/Abstract])) OR (random forest[Title/Abstract])) OR (neural network[Title/Abstract])) OR (neural networks[Title/Abstract])) OR (K-Nearest Neighbor[Title/Abstract])) OR (CNN[Title/Abstract])) OR (Support vector machine[Title/Abstract])) OR (SVM[Title/Abstract])) OR (Gradient Boosting Machine[Title/Abstract])) OR (Nomogram[Title/Abstract])) OR (XGBoost[Title/Abstract])) OR (Adaboost[Title/Abstract])) OR (Decision tree[Title/Abstract])) OR (ResNet-50[Title/Abstract])) OR (ResNet[Title/Abstract])) OR (Naive Bayesian[Title/Abstract])) OR (Multilayer perceptron[Title/Abstract])) OR (Bayesian network[Title/Abstract])) OR (Radiomics[Title/Abstract])) OR (Radiomic[Title/Abstract])) OR (Prediction model[Title/Abstract])) OR (Risk model[Title/Abstract]) | 295,437 |
| #6 | (machine learning[MeSH Terms]) OR (((((((((((((((((((((((((Transfer Learning[Title/Abstract]) OR (Deep learning[Title/Abstract])) OR (Ensemble Learning[Title/Abstract])) OR (artificial intelligence[Title/Abstract])) OR (random forest[Title/Abstract])) OR (neural network[Title/Abstract])) OR (neural networks[Title/Abstract])) OR (K-Nearest Neighbor[Title/Abstract])) OR (CNN[Title/Abstract])) OR (Support vector machine[Title/Abstract])) OR (SVM[Title/Abstract])) OR (Gradient Boosting Machine[Title/Abstract])) OR (Nomogram[Title/Abstract])) OR (XGBoost[Title/Abstract])) OR (Adaboost[Title/Abstract])) OR (Decision tree[Title/Abstract])) OR (ResNet-50[Title/Abstract])) OR (ResNet[Title/Abstract])) OR (Naive Bayesian[Title/Abstract])) OR (Multilayer perceptron[Title/Abstract])) OR (Bayesian network[Title/Abstract])) OR (Radiomics[Title/Abstract])) OR (Radiomic[Title/Abstract])) OR (Prediction model[Title/Abstract])) OR (Risk model[Title/Abstract])) | 315,419 |
| #7 | Suicide[MeSH Terms] | 76,971 |
| #8 | (((((((Suicides[Title/Abstract]) OR (Suicidal[Title/Abstract])) OR (Assisted Death[Title/Abstract])) OR (Assisted Deaths[Title/Abstract])) OR (Parasuicide[Title/Abstract])) OR (Parasuicides[Title/Abstract])) OR (Fatal Attempt[Title/Abstract])) OR (Fatal Attempts[Title/Abstract]) | 48,300 |
| #9 | (Suicide[MeSH Terms]) OR ((((((((Suicides[Title/Abstract]) OR (Suicidal[Title/Abstract])) OR (Assisted Death[Title/Abstract])) OR (Assisted Deaths[Title/Abstract])) OR (Parasuicide[Title/Abstract])) OR (Parasuicides[Title/Abstract])) OR (Fatal Attempt[Title/Abstract])) OR (Fatal Attempts[Title/Abstract])) | 93,482 |
| #10 | ((("Adolescent"[Mesh]) OR ((((((((((((((Adolescents[Title/Abstract]) OR (Adolescence[Title/Abstract])) OR (Teens[Title/Abstract])) OR (Teen[Title/Abstract])) OR (Teenagers[Title/Abstract])) OR (Teenager[Title/Abstract])) OR (Youth[Title/Abstract])) OR (Youths[Title/Abstract])) OR (child[Title/Abstract])) OR (youth[Title/Abstract])) OR (juvenile[Title/Abstract])) OR (children[Title/Abstract])) OR (minor[Title/Abstract])) OR (minors[Title/Abstract]))) AND ((machine learning[MeSH Terms]) OR (((((((((((((((((((((((((Transfer Learning[Title/Abstract]) OR (Deep learning[Title/Abstract])) OR (Ensemble Learning[Title/Abstract])) OR (artificial intelligence[Title/Abstract])) OR (random forest[Title/Abstract])) OR (neural network[Title/Abstract])) OR (neural networks[Title/Abstract])) OR (K-Nearest Neighbor[Title/Abstract])) OR (CNN[Title/Abstract])) OR (Support vector machine[Title/Abstract])) OR (SVM[Title/Abstract])) OR (Gradient Boosting Machine[Title/Abstract])) OR (Nomogram[Title/Abstract])) OR (XGBoost[Title/Abstract])) OR (Adaboost[Title/Abstract])) OR (Decision tree[Title/Abstract])) OR (ResNet-50[Title/Abstract])) OR (ResNet[Title/Abstract])) OR (Naive Bayesian[Title/Abstract])) OR (Multilayer perceptron[Title/Abstract])) OR (Bayesian network[Title/Abstract])) OR (Radiomics[Title/Abstract])) OR (Radiomic[Title/Abstract])) OR (Prediction model[Title/Abstract])) OR (Risk model[Title/Abstract])))) AND ((Suicide[MeSH Terms]) OR ((((((((Suicides[Title/Abstract]) OR (Suicidal[Title/Abstract])) OR (Assisted Death[Title/Abstract])) OR (Assisted Deaths[Title/Abstract])) OR (Parasuicide[Title/Abstract])) OR (Parasuicides[Title/Abstract])) OR (Fatal Attempt[Title/Abstract])) OR (Fatal Attempts[Title/Abstract]))) | 159 |

**2.Cochrane**

| Search number | Query | Results |
| --- | --- | --- |
| #1 | MeSH descriptor: [Adolescent] explode all trees | 136681 |
| #2 | (Adolescent):ti,ab,kw OR (Adolescents):ti,ab,kw OR (Adolescence):ti,ab,kw OR (Teens):ti,ab,kw OR (Teen):ti,ab,kw | 173367 |
| #3 | (Teenagers):ti,ab,kw OR (Teenager):ti,ab,kw OR (Youth):ti,ab,kw OR (Youths):ti,ab,kw OR (child):ti,ab,kw | 193680 |
| #4 | (youth):ti,ab,kw OR (juvenile):ti,ab,kw OR (children):ti,ab,kw OR (minor):ti,ab,kw OR (minors):ti,ab,kw | 211472 |
| #5 | #1 or #2 or #3 or #4 | 319909 |
| #6 | MeSH descriptor: [Machine Learning] explode all trees | 975 |
| #7 | (machine learning):ti,ab,kw OR (Transfer Learning):ti,ab,kw OR (Deep learning):ti,ab,kw OR (Ensemble Learning):ti,ab,kw OR (artificial intelligence):ti,ab,kw | 7210 |
| #8 | (random forest):ti,ab,kw OR (neural network):ti,ab,kw OR (neural networks):ti,ab,kw OR (K-Nearest Neighbor):ti,ab,kw OR (CNN):ti,ab,kw | 4497 |
| #9 | (Support vector machine):ti,ab,kw OR (SVM):ti,ab,kw OR (Gradient Boosting Machine):ti,ab,kw OR (Nomogram):ti,ab,kw OR (XGBoost):ti,ab,kw | 2467 |
| #10 | (Adaboost):ti,ab,kw OR (Decision tree):ti,ab,kw OR (ResNet-50):ti,ab,kw OR (ResNet):ti,ab,kw OR (Naive Bayesian):ti,ab,kw | 1130 |
| #11 | (Multilayer perceptron):ti,ab,kw OR (Bayesian network):ti,ab,kw OR (Radiomics):ti,ab,kw OR (Radiomic):ti,ab,kw OR (Prediction model):ti,ab,kw | 7010 |
| #12 | (Risk model):ti,ab,kw | 31784 |
| #13 | #6 or #7 or #8 or #9 or #10 or #11 or #12 | 45731 |
| #14 | MeSH descriptor: [Suicide] explode all trees | 0 |
| #15 | (Suicide):ti,ab,kw OR (Suicides):ti,ab,kw OR (Suicidal):ti,ab,kw OR (Assisted Death):ti,ab,kw OR (Assisted Deaths):ti,ab,kw | 10114 |
| #16 | (Parasuicide):ti,ab,kw OR (Parasuicides):ti,ab,kw OR (Fatal Attempt):ti,ab,kw OR (Fatal Attempts):ti,ab,kw | 181 |
| #17 | #14 or #15 or #16 | 10215 |
| #18 | #5 and #13 and #17 | 191 |

**3.Embase**

| Search number | Query | Results |
| --- | --- | --- |
| #1 | 'adolescent'/exp | 1981284 |
| #2 | adolescent:ab,ti OR adolescents:ab,ti OR adolescence:ab,ti OR teens:ab,ti OR teen:ab,ti OR teenagers:ab,ti OR teenager:ab,ti OR youths:ab,ti OR child:ab,ti OR youth:ab,ti OR juvenile:ab,ti OR children:ab,ti OR minor:ab,ti OR minors:ab,ti | 2765366 |
| #3 | #1 OR #2 | 3968742 |
| #4 | 'machine learning'/exp | 470838 |
| #5 | 'machine learning':ab,ti OR 'transfer learning':ab,ti OR 'deep learning':ab,ti OR 'ensemble learning':ab,ti OR 'artificial intelligence':ab,ti OR 'random forest':ab,ti OR 'neural network':ab,ti OR 'neural networks':ab,ti OR 'k-nearest neighbor':ab,ti OR cnn:ab,ti OR 'support vector machine':ab,ti OR svm:ab,ti OR 'gradient boosting machine':ab,ti OR nomogram:ab,ti OR xgboost:ab,ti OR adaboost:ab,ti OR 'decision tree':ab,ti OR 'resnet 50':ab,ti OR resnet:ab,ti OR 'naive bayesian':ab,ti OR 'multilayer perceptron':ab,ti OR 'bayesian network':ab,ti OR radiomics:ab,ti OR radiomic:ab,ti OR 'prediction model':ab,ti OR 'risk model':ab,ti | 400208 |
| #6 | #4 OR #5 | 634037 |
| #7 | 'suicide'/exp | 72275 |
| #8 | suicide:ab,ti OR suicides:ab,ti OR suicidal:ab,ti OR 'assisted death':ab,ti OR parasuicide:ab,ti OR parasuicides:ab,ti OR 'fatal attempt':ab,ti OR 'fatal attempts':ab,ti OR 'assisted deaths':ab,ti | 125782 |
| #9 | #7 OR #8 | 144478 |
| #10 | #3 AND #6 AND #9 | 322 |

**4.Web of science**

| Search number | Query | Results |
| --- | --- | --- |
| #1 | TS=(Adolescent OR Adolescents OR Adolescence OR Teens OR Teen OR Teenagers OR Teenager OR Youth OR Youths OR child OR youth OR juvenile OR children OR minor OR minors) | 3091996 |
| #2 | TS=(machine learning OR Transfer Learning OR Deep learning OR Ensemble Learning OR artificial intelligence OR random forest OR neural network OR neural networks OR K-Nearest Neighbor OR CNN OR Support vector machine OR SVM OR Gradient Boosting Machine OR Nomogram OR XGBoost OR Adaboost OR Decision tree OR ResNet-50 OR ResNet OR Naive Bayesian OR Multilayer perceptron OR Bayesian network OR Radiomics OR Radiomic OR Prediction model OR Risk model) | 3200683 |
| #3 | TS=(Suicide OR Suicides OR Suicidal OR Assisted Death OR Assisted Deaths OR Parasuicide OR Parasuicides OR Fatal Attempt OR Fatal Attempts) | 148795 |
| #4 | #1 AND #2 AND #3 | 4060 |
