# Supplementary figures and images for: Predictive Performance of Machine Learning for Suicide in Adolescents: Systematic Review and Meta-Analysis
Source: J Med Internet Res. 2025 Jun 16;27:e73052. doi: 10.2196/73052 (PMC12209725; doi:10.2196/73052)

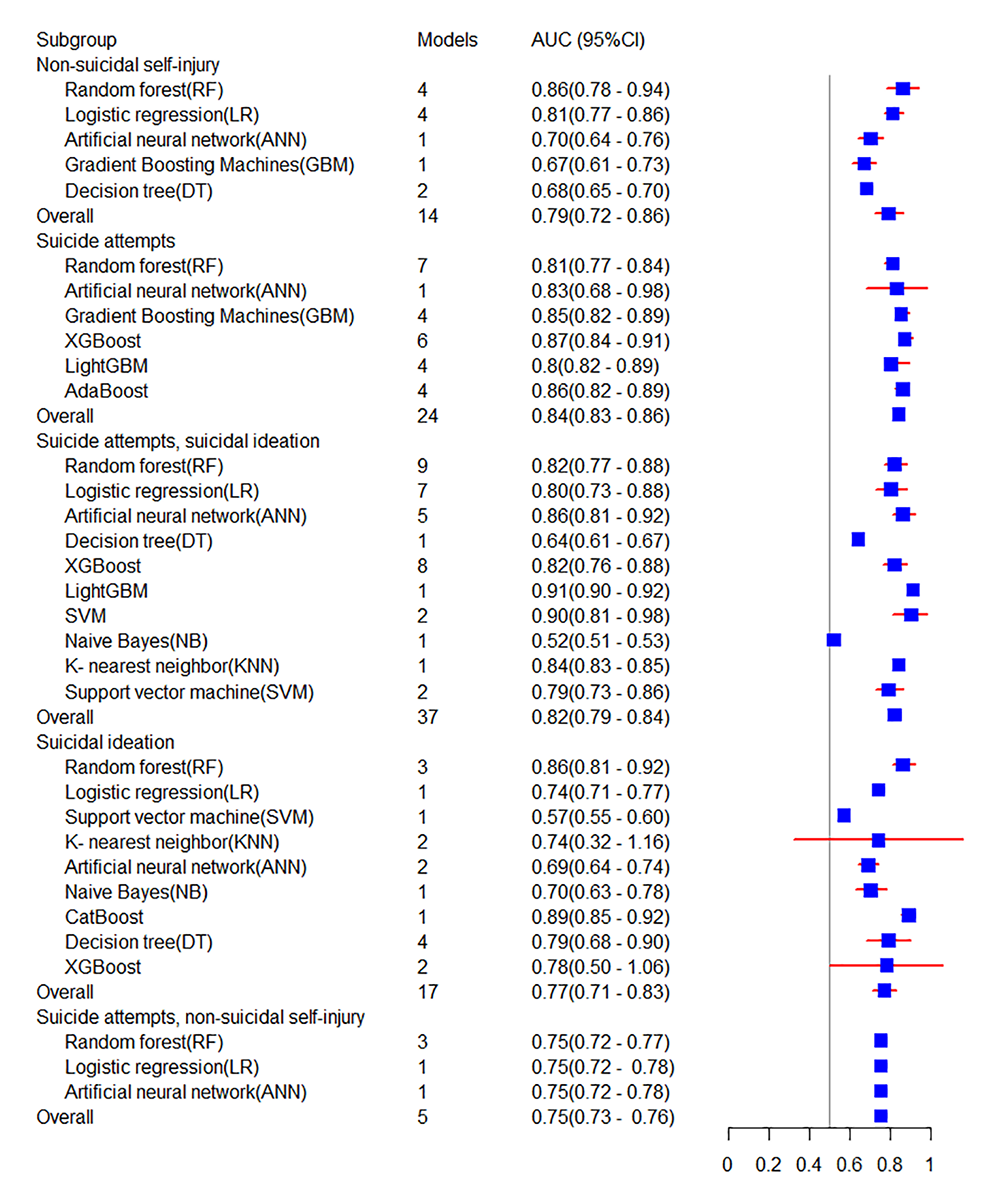

Supplement: Multimedia Appendix 3 [file jmir_v27i1e73052_app3.png]
